# Methods for peptide identification by spectral comparison

**DOI:** 10.1186/1477-5956-5-3

**Published:** 2007-01-16

**Authors:** Jian Liu, Alexander W Bell, John JM Bergeron, Corey M Yanofsky, Brian Carrillo, Christian EH Beaudrie, Robert E Kearney

**Affiliations:** 1 Center for Cellular & Biomolecular Research, University of Toronto, Toronto, Canada; 2 Montreal Proteomics Network, Montreal, Canada; 3 Department of Anatomy and Cell Biology, McGill University, Montreal, Canada; 4 Department of Biomedical Engineering, McGill University, Montreal, Canada

## Abstract

**Background:**

Tandem mass spectrometry followed by database search is currently the predominant technology for peptide sequencing in shotgun proteomics experiments. Most methods compare experimentally observed spectra to the theoretical spectra predicted from the sequences in protein databases. There is a growing interest, however, in comparing unknown experimental spectra to a library of previously identified spectra. This approach has the advantage of taking into account instrument-dependent factors and peptide-specific differences in fragmentation probabilities. It is also computationally more efficient for high-throughput proteomics studies.

**Results:**

This paper investigates computational issues related to this spectral comparison approach. Different methods have been empirically evaluated over several large sets of spectra. First, we illustrate that the peak intensities follow a Poisson distribution. This implies that applying a square root transform will optimally stabilize the peak intensity variance. Our results show that the square root did indeed outperform other transforms, resulting in improved accuracy of spectral matching. Second, different measures of spectral similarity were compared, and the results illustrated that the correlation coefficient was most robust. Finally, we examine how to assemble multiple spectra associated with the same peptide to generate a synthetic reference spectrum. Ensemble averaging is shown to provide the best combination of accuracy and efficiency.

**Conclusion:**

Our results demonstrate that when combined, these methods can boost the sensitivity and specificity of spectral comparison. Therefore they are capable of enhancing and complementing existing tools for consistent and accurate peptide identification.

## Background

One key issue in proteomics is to identify proteins and characterize their expressions in cells. Tandem mass spectrometry paired with advanced liquid chromatography has emerged as the standard technique for high throughput protein identification [1,2]. This shotgun technology does not require the initial separation of individual proteins and therefore can be applied to complex mixtures. Typically, a tissue sample is first fractionated, the resulting mixture of proteins is digested into peptides by an enzyme such as trypsin. The peptide mixture is then separated by High Performance Liquid Chromatography (HPLC), ionized and sent to a mass spectrometer to measure the mass/charge ratio of each peptide. Peptides of interest are selected for further fragmentation in a collision cell to produce tandem (MS/MS) mass spectra. A MS/MS spectrum consists of a sequence of peaks, each characterizing the mass/charge ratio and intensity of an ion. Computer software is then used to identify the peptide sequence associated with each MS/MS spectrum. Finally, the identified peptides are grouped together to determine the underlying proteins.

Historically, methods for identifying peptides from MS/MS spectra can be categorized into two general classes. In the first group, *De Novo *sequencing methods such as PEAKS [3] reconstruct the peptide sequence from the spectrum based on knowledge of the peptide fragmentation pattern. This class of algorithms requires high quality spectra with nearly complete ladders of *b/y *ions. The second method is database search. This approach compares the experimental spectrum against theoretical spectra determined by the *in silico *digestion and fragmentation of known proteins in a sequence database. This approach is currently preferred due to its reliability in practice. MASCOT [4] and SEQUEST [5] are examples of this approach which employ sophisticated statistical models to determine the similarity of experimental and theoretical spectra. Some recent studies [6,7] further proposed the combination of the two approaches by using sequence tags, determined by *De Novo *to reduce the number of peptides during the database search.

Peptide fragmentation is a complex process, so spectra generated from mass spectrometers are often significantly different from their theoretical counterparts. As a result, sophisticated algorithms, such as dynamic programming [3] and Hidden Markov Models [8], have been used in the kernel of database search methods to recognize spectra in such circumstances. These algorithms require very intense computations so that the search time becomes a bottleneck, especially when high-throughput experiments produce hundreds of thousands of spectra in hours. This problem is exacerbated when the possibility of unknown post-translational modifications (PTMs) must be considered [9]. It not only drastically increases computations required for a blind PTM search, but also severely jeopardizes the discovery of true positives. To cope with these challenges, peptide identification by direct comparison of experimental spectra has drawn much attention. This approach has the advantage of taking into account instrument-dependent or peptide-specific contributing factors for producing tandem spectra. Moreover, duplicate spectra are ubiquitous in large scale proteomic data [10] due to the following facts: 1) many proteins may share the same peptides; 2) the same peptides may be fragmented multiple times or repeated in different runs. It would also reduce the search time substantially by recognizing the duplicates. Therefore, spectral comparison provides another means to support high throughput peptide sequencing with current techniques [11-16]. The software package NoDupe [10] was designed to detect duplicate peptide spectra. Other tools, such as Pep-Miner [17] and MS2grouper [18], cluster spectra by their similarity and derive a representative spectrum. Tools of this type allow comparison of the protein/peptide contents of different sample mixtures without the actual identification of peptides. Recently, software tools X! Hunter [19] and BiblioSpec [20] have been available in the public domain for searching libraries and compiling spectra. In this study, we examined some computational issues related to the identification of peptides by spectral comparison. Our goal was to explore the methods to identify peptides by comparing experimental spectra to a library of reference spectra of known origin. We first surveyed related studies and conducted a comparative study on fundamental issues of spectral comparison. These include how to normalize peak intensities, vectorize spectra, and measure their pairwise similarity. We then investigated how to construct a robust representative spectrum from a group of spectra produced from the same peptide.

## Results and discussion

In this section, we present the experimental results for peptide identification by evaluating the spectral similarity. Detailed specifications of some terms and experimental spectra are presented in the Methods section.

### Experimental results

**Figure 1:**
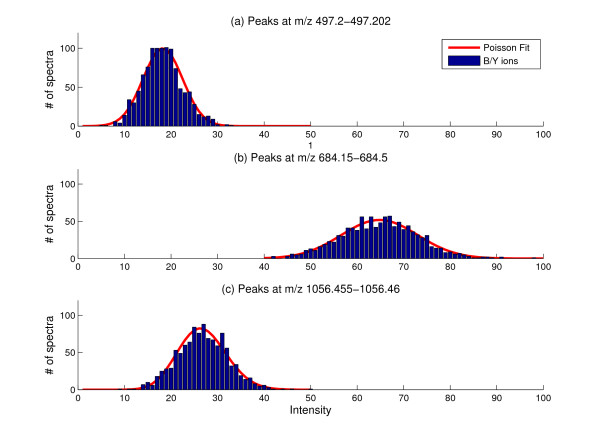
The peak intensities in GluFib MS/MS spectra can be approximated by a Poisson distribution. Histograms of peak intensities observed in sequential ms/ms runs are shown. The superimposed fitting curves are those predicted for the Poisson distributions. Each panel illustrates the result for peaks at a different m/z value.

As described in the Methods section, it is assumed that peak intensities follow Poisson distribution. We first verified this hypothesis as the basis for further optimization. About 1,000 spectra were generated by the direct infusion of the peptide GluFib (peptide sequence EGVNDNEEGFFSAR) during the calibration of the mass spectrometer. Fig. 1 shows the distribution of ion intensities recorded at three different m/z values, fitted by the theoretical Poisson distributions. Although the peak intensities for different ions have different mean values and variability, a Poisson distribution provides a close approximation for each of these ions. This enables us to stabilize the peak intensity by square root transform.

In this study, three datasets were used to examine the effectiveness of our proposed methods. Details on the spectra can be found in the Datasets section. For these datasets, the mass errors of different spectra assigned to the same peptides were generally very small, and over 90% of them were less than 0.1 Da. During profile generation, the bin size and error window were set at 1 and 0.1 Da, respectively. Since spectra from the same peptide with different charge states may be very different [21], spectra were compared only when their precursor ion masses were within 2 Da error tolerance and with the same charge states.

**Figure 2:**
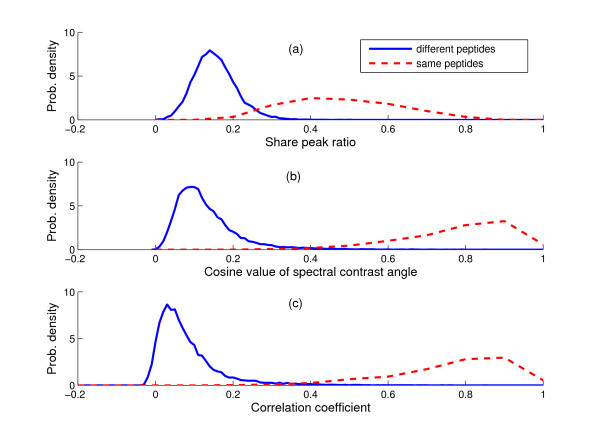
Distributions of scores for different similarity measures for spectra (dataset 1) from the same and different peptides: (a) shared peak ratio, (b) cosine value and (c) correlation coefficient.

All the experimental spectra were previously matched to peptides using MASCOT. These peptide identifications were used to cluster the spectra into groups matching the same peptide. Ideally the pairwise similarity score should be high for two spectra from the same peptide but low for spectra from different peptides. To verify this assumption, we examined the distributions of similarity scores for spectra assigned to the same and different peptides for various configurations. These two score populations were denoted as *P*_*ss *_and *P*_*sd*_, respectively. Experiments were conducted to examine the effectiveness of different similarity measures of pairwise spectral comparison. Fig. 2 shows the distributions of three similarity scores generated from dataset 1: ratio of shared peaks, cosine correlation value, and correlation coefficient. It is evident that there is a greater separation between *P*_*ss *_and *P*_*sd *_for the cosine and correlation measures than for the shared peak ratio. This suggests that they would provide a better basis for comparing spectra. Correlation coefficients appeared to be a little more robust than the cosine values since the *P*_*ss *_values tended to be smaller. Therefore, the correlation coefficient was used as the similarity measure in the remainder of this work.

**Figure 3:**
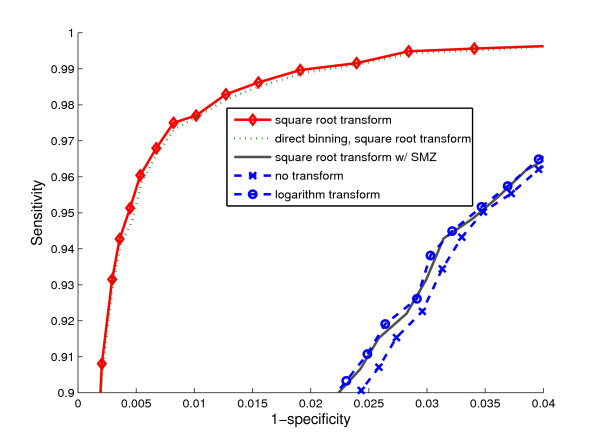
ROC curves obtained for spectral comparison (dataset 1) using different signal transforms. The square root transform performs significantly better than other methods.

The selection of a similarity measure and the way reference spectra are modeled constitute a clustering configuration. To assess the effects of the different factors in clustering spectra, we tested eight typical clustering configurations over dataset 1. Table 1 specifies the details of each configuration. Spectra matched to the same peptide were clustered together. A total of 585 clusters were constructed, varying in size from 2 to 19 spectra. A reference spectrum was constructed for each clustering configuration, except for the closest neighbor scheme where all spectra served as representatives in turn during the matching process. Each experimental spectrum was then compared to all references within error tolerance of precursor mass. The spectrum was assigned to the peptide associated with the reference with the highest similarity scores. Finally, we examined whether the assignments were consistent with those of the MASCOT identifications. Assuming that the MASCOT results were correct, Receiver Operating Characteristic (ROC) curves were then computed for each configuration. Their performances can be evaluated by the areas under the ROC curves, where a larger area usually indicates better performance. The eight configurations in Table 1 were designed to unveil the impact of various intensity transforms and reference modeling on spectral comparison. Fig. 3 shows the effectiveness of the different intensity transforms. The best performance was obtained with the two configurations in which the spectra were transformed by the square root prior to comparison. The overall performance of these configurations was substantially better than those obtained with no transform, the logarithm transform, or by weighting the intensity with the square of the m/z ratio (SMZ). Thus, the variance stabilization property of the square root transform improved the performance. It is apparent that profiling-based comparison was only slightly better than that of direct binning. We attribute this to the fact that the spectra were produced by high resolution QTOF. The B/Y peaks in the spectra of the same peptides were well aligned; their m/z errors were generally smaller than 0.1 Da. Consequently, only 1% of the peaks were allocated to neighboring bins in spectral profiles. For low accuracy instruments with larger m/z errors, we anticipate that the profiling technique will perform more robustly.

**Table 1:** Specification of eight typical configurations for spectral comparison. (For pairwise comparison in 1–5, the experimental spectra with the highest scores were selected as references from the clusters. SMZ in row 4 indicates that the intensity is weighted by the square of m/z as in [20].)

Setting ID	Intensity transform	Binning method	Reference spectra	ROC area
1	logarithm	profiling	individual spectra	0.993
2	no transform	profiling	individual spectra	0.992
3	square root w/SMZ	profiling	individual spectra	0.993
4	square root	direct binning	individual spectra	0.997
5	square root	profiling	individual spectra	0.998
6	square root	profiling	theoretical spectra	0.987
7	square root	profiling	closest neighbors	0.999
8	square root	profiling	average spectra	0.999

**Figure 4:**
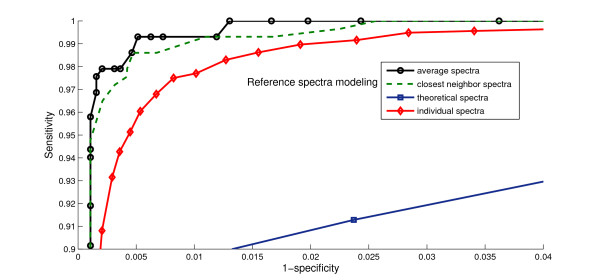
Effects of different models of reference spectra (dataset 1) on the ROC curves. Ensemble average and closest neighbor perform better than other methods.

Fig. 4 further illustrates how the choice of the reference spectra influenced sensitivity and specificity. The best performance was obtained using the average spectra as the references. The closest neighbors configuration performed almost as well as the average spectra. However, it is much more computationally efficient to use the average spectra since it requires only a single pairwise comparison for each candidate peptide. Both of them performed somewhat better than using the individual experimental spectra as the reference. The ROC curve for theoretical spectra was much worse than the other three. This observation suggests that peptide and instrument-specific factors play a significant role in the direct spectral comparison.

### Generalizing the performance of reference spectra

As stated earlier, the error tolerance of the precursor mass was set to 2 Da for pairwise comparison during the experiments. In our dataset there were typically only a few spectra from different peptides within this tolerance range. However, for large scale proteomics studies, an experimental spectrum may have to be compared with many reference spectra. To assess the performance expected under such circumstances, we ran a Monte Carlo simulation for different numbers of candidate peptides. The sensitivity and specificity for a cutoff similarity score *s*_*c *_was calculated as follows. We assume that there are *k *reference spectra within the mass error tolerance window for a given spectrum. Assign the spectrum to the reference spectrum with the highest spectral similarity measure above the threshold *s*_*c*_. There are two possibilities: either the given spectrum is generated from the peptide associated with the reference spectrum or not. The sensitivity and specificity can be determined from either possibility. In the first case, one score *s *was randomly drawn from *P*_*ss*_, and the remaining *k *- 1 scores {*d*_1_,..., *d*_*k*-1_} were drawn from *P*_*sd*_. Repeating this procedure for a sufficient number of times determines the sensitivity as the frequency of *s *≥ *s*_*c *_and *s *≥ *d*_*i*_, where *i *= 1,... *k *- 1. Similarly, in the second case, *k *scores {*d*_1_,..., d_*k*_} from *P*_*sd *_were drawn at each repeat. Specificity is then approximated by the frequency of *s*_*c *_≥ *d*_*i*_, where *i *= 1,..., *k*.

Table 2 shows the ROC areas using average and individual spectra as references when the number of candidate spectra increases. It is clear that the average spectra as the reference consistently outperformed individual spectra. Its advantage becomes more pronounced when the number of candidate peptides is large.

**Table 2:** Performance comparison using average and individual spectra as references

# of candidate peptides	ROC area	
	
	Average spectra	Individual spectra
10	0.993	0.983
100	0.942	0.900
500	0.826	0.762
1,000	0.764	0.685

According to the traditional academic point system, a classifier is considered to be excellent or good when the area under its ROC curve is in the range of [0.9,1] or [0.8,0.9]. We noticed that when the number of candidate peptides reaches 1,000, the spectral comparison does not provide a good balance of sensitivity and specificity. This is a common drawback for statistical measures of spectral similarity. For instance, to ensure satisfactory sensitivity, SEQUEST [5] only compares the cross correlation of the top 500 peptides produced from the preliminary selection stage. In practice, however, the database search is usually limited to a specific taxonomy. Since the number of candidate peptides is relatively small after the search scope is narrowed down on the basis of parent ion mass, this method will provide adequate sensitivity for spectral comparison.

### Further validation

To determine how well the above results are reproducible, we cross-examined spectra from datasets 1 and 2. Within each dataset, spectra assigned to the same peptides were clustered. There were 537 clusters of size 2, 255 clusters of size 3, and 388 clusters with more than 3 spectra. Their average spectra were also constructed. As described in the Datasets section, these two datasets shared many peptides. For each pairwise comparison, an individual and a reference spectra were drawn from different datasets. Score distributions similar to those in Fig. 2 were generated. This reproducibility confirmed the robustness of our approach for spectral comparison.

**Figure 5:**
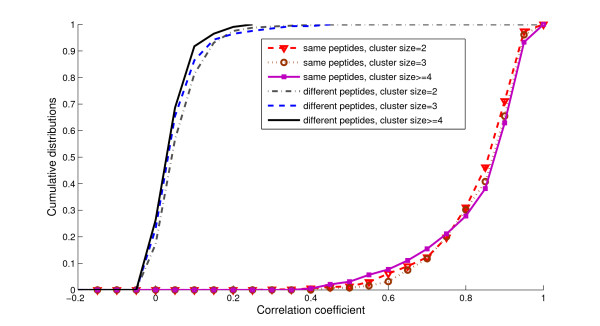
Cross validation between datasets 1 and 2: cumulative distributions of similarity scores for the same and different peptides in spectrum clusters of different sizes. Increasing the sizes of clusters leads to better separation of spectral similarity scores between the same and different peptides.

One intriguing question is how the cluster sizes influence the performance of average spectra. To answer this question, the similarity scores are further partitioned based on the cluster sizes. Fig. 5 depicts the cumulative distributions of similarity scores. As the cluster size increased, the similarity between references and spectra from different peptides tended to decrease, while their similarity to those of the same peptides increased. Therefore, increasing the cluster sizes helps to enhance the performance asymptotically while the noisy peaks diminish gradually after averaging.

**Figure 6:**
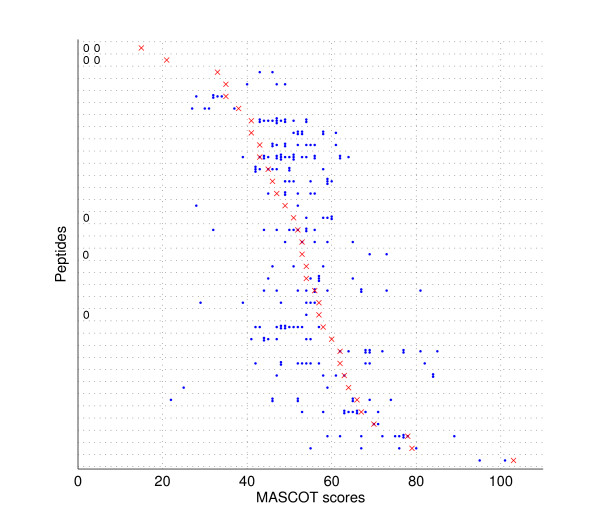
MASCOT Scores for the individual and average spectra from the 35 peptides in dataset 3, sorted by the MASCOT score of the ensemble average spectrum. MASCOT scores of individual spectra are indicated by blue dots ("0" indicates that the spectrum was not identified by MASCOT); those of average spectra are marked by red crosses.

Dataset 3 was used to further examine the quality of ensemble average spectra. The original spectra were annotated by searching against customized protein databases for specific taxonomies. Based on their initial annotations, we derived 35 clusters, whose average spectra were also constructed. The individual and the average spectra were searched against the NCBInr database using the MASCOT online server. Fig. 6 shows the MASCOT scores for individual and average spectra. Note that the initial peptide identifications were based upon a search against smaller customized protein databases, therefore some spectra were not recognized when the search space increased to the whole NCBInr database. Fig. 6 illustrates that all the average spectra were correctly recognized. Moreover, for two clusters, the average spectra were identified even though original spectra failed MASCOT. In other words, the average spectra generally retained the quality to be identifiable since the peak amplitude of noise after averaging was decreased.

## Conclusion

Database search is currently the prevailing approach to sequence peptides from MS/MS spectra. However, this approach is often compromised by a large number of unassigned spectra because the fragmentation process is both peptide-specific and instrument-dependent. On the other hand, it is very computationally expensive to analyze spectra when the peptides have undergone unknown modifications and mutations or the protein database is very large. Current tandem mass spectrometry has standardized the conditions used for acquiring data, therefore similar experimental spectra are highly reproducible for the same peptides. This enables the direct comparison of experimental spectra as an alternative method. Some recent studies X! Hunter [19] and BiblioSpec [20] have demonstrated that it is practically feasible to construct representatives from spectra, even they were generated by different brands and types of instruments. A pairwise comparison of spectra can be finished within tens of milliseconds; it is much faster than a traditional database search that can take up to a few minutes on a modern computer. Therefore, it provides an efficient alternative to identify peptides via tandem mass spectra.

This paper systematically examined some methodological and computational issues related to this approach. Our experimental results confirmed that transforming intensity signals by the square root stabilizes variance and therefore facilitates spectral comparison. Also, statistical measures such as correlation coefficients can provide a good indication of their pairwise similarity. In contrast to other studies focusing on optimizing peak selection to construct representative spectra, we discovered that ensemble averaging spectra is an effective and efficient way to generate reference spectra. This method outperformed the use of individual experimental spectra as a reference, and the advantage increased as the candidate peptide set became large.

In summary, our methods and results provide a sound basis to further develop algorithms to optimize peptide identification by spectral comparison.

## Methods

In this section, we first describe the spectra used in our experiments. We then address the computational issues related to spectral comparison at different levels. These subjects include how to adjust peak intensity from raw spectra and vectorize spectral data, the measurement of spectral similarity, and the methods to build up the reference spectra.

### Datasets

In our experiments, three datasets of tandem mass spectra were used to validate our methods. Two of them were obtained from highly enriched rat liver organelle (endoplasmic reticulum) fractions provided by McGill University and the University of Montreal. Organelle collection, purification, and characterization were carried out as previously described [22-24]. Organelle proteins were separated using 1D-SDS PAGE, in-gel trypsinized, excised, and analyzed by an LC QTOF mass spectrometer, using standard methods [25,26]. Raw tandem MS data were processed using MASCOT Distiller for peak detection, and processed spectra were searched against the NCBInr database with taxonomy restricted to rattus (30,949 protein sequences) using MASCOT Cluster v 1.9.03 software [27]. The spectra along with the MASCOT database search results were then stored in an in-house Cellmap database. More details on the experimental protocols and spectra can be found in [28].

The first dataset was obtained from the analysis of the entire fraction of the endoplasmic reticulum rough membrane. Initially the dataset had 8,323 spectra, but after removing the spectra with negative MASCOT scores (the difference between the identity score and peptide score for a given spectrum), only 5,454 spectra were left, leading to the identification of 3,364 unique peptides. The second set of spectra was used for further cross validation. These data were acquired with the same procedures as the first dataset, except that the sample was the detergent fraction of the endoplasmic reticulum rough membrane. Initially this dataset was composed of 13,966 spectra. Only 4,051 spectra were left after removing the spectra with negative MASCOT scores. 3,825 unique peptides were identified from these spectra. Since these two datasets were produced from the same organelle, there were 1,173 peptides common for both benchmarks. The third dataset was the benchmark data in our previous work [29], which was acquired from other research institutes. It consisted of 367 spectra generated by LCQ/QTOF instruments, and the peptides were identified by the MASCOT search engine against proprietary databases and further validated by experts. Many of these spectra mapped to the same peptides, finally we constructed 35 clusters.

### Peak intensity transforms

It is well known that the magnitude of peak intensities in MS/MS spectra can vary by several orders of magnitude. In order to neutralize this influence on spectral comparison, it should be advantageous to transform the peak intensity in certain ways prior to comparison. Previous studies have suggested using square root (such as NoDupe [10] and [30]), the logarithm (PEAKS [3]) for the transform, or weighing the intensity by square of m/z (SMZ) as in BiblioSpec [20].

In particular, QTOF instruments employ time-to-digital converters (TDCs) to record the abundance of ions. TDCs divide time into bins and register the bin when ion detection events occur during scan cycles. As a result, peak intensities generated can be expected to follow a Poisson distribution [31]. If this is the case, the standard deviation of the peak intensities will vary with the mean value. In this situation, the square root transform may be the optimal transform since the variance of the transformed variable will be constant. To understand why, assume the original intensity *I *has mean *λ *and variance *λ*. If I* = I
 MathType@MTEF@5@5@+=feaafiart1ev1aaatCvAUfKttLearuWrP9MDH5MBPbIqV92AaeXatLxBI9gBaebbnrfifHhDYfgasaacH8akY=wiFfYdH8Gipec8Eeeu0xXdbba9frFj0=OqFfea0dXdd9vqai=hGuQ8kuc9pgc9s8qqaq=dirpe0xb9q8qiLsFr0=vr0=vr0dc8meaabaqaciaacaGaaeqabaqabeGadaaakeaadaGcaaqaaiabdMeajbWcbeaaaaa@2DE2@, then by using a first-order Taylor expansion, we have I* ≈ λ
 MathType@MTEF@5@5@+=feaafiart1ev1aaatCvAUfKttLearuWrP9MDH5MBPbIqV92AaeXatLxBI9gBaebbnrfifHhDYfgasaacH8akY=wiFfYdH8Gipec8Eeeu0xXdbba9frFj0=OqFfea0dXdd9vqai=hGuQ8kuc9pgc9s8qqaq=dirpe0xb9q8qiLsFr0=vr0=vr0dc8meaabaqaciaacaGaaeqabaqabeGadaaakeaadaGcaaqaaGGaciab=T7aSbWcbeaaaaa@2E82@ + (*I *- *λ*) * 0.5 * *λ*^-0.5^. Therefore,

*Variance*(I*) ≈ *Variance*(λ
 MathType@MTEF@5@5@+=feaafiart1ev1aaatCvAUfKttLearuWrP9MDH5MBPbIqV92AaeXatLxBI9gBaebbnrfifHhDYfgasaacH8akY=wiFfYdH8Gipec8Eeeu0xXdbba9frFj0=OqFfea0dXdd9vqai=hGuQ8kuc9pgc9s8qqaq=dirpe0xb9q8qiLsFr0=vr0=vr0dc8meaabaqaciaacaGaaeqabaqabeGadaaakeaadaGcaaqaaGGaciab=T7aSbWcbeaaaaa@2E82@ + (*I *- *λ*) * 0.5 * *λ*^-0.5^)

≈ 0.25 * *λ*^-1 ^* *Variance*(*I*)

≈ 0.25     (1)

In other words, after applying the square root transform, the variance of the peak intensities is stabilized at approximately 0.25. Therefore, this transform is applied to the experimental spectra as a preprocessing step in our experiments, unless otherwise stated.

### Profiling spectra

Spectral comparison can be performed in a number of ways. Some approaches match spectra based on the similarity of individual peaks [5,17,32]. Another approach is to vectorize the whole spectrum, and then calculate the distance between two vectors. Here, the peak list of a spectrum is evenly divided into a consecutive sequence of bins on the *m/z *axis, and a vector for the spectrum is derived by summing up the intensities of peaks in each bin. This method has been used in many studies [10,18], and we refer to it as direct binning. However, as pointed out in [19,32], it is not straightforward to establish the correspondence between peaks and bins. The measured *m/z *value of a peak is subject to measurement errors; in other words, its theoretical counterpart can be either larger or smaller.

To avoid the above pitfall, we used an enhanced profiling technique that reduces the problem of irregular sampling of mass spectra. For simplicity, it is assumed that *m/z *values following a uniform distribution in an error window. During the profiling step, the intensity of each peak is distributed into neighboring bins. Formally, given the bin width *w *and a *m/z *error window *e*, and assume that *w *≥ *e *and a peak with value *m *for m/z ratio is located inside *b *- *th *bin [*l,r*], then its intensity *i *is proportioned into three consecutive bins as follows:

Ib−1=i∗l−min(l,m−0.5∗e)e     (2)
 MathType@MTEF@5@5@+=feaafiart1ev1aaatCvAUfKttLearuWrP9MDH5MBPbIqV92AaeXatLxBI9gBaebbnrfifHhDYfgasaacH8akY=wiFfYdH8Gipec8Eeeu0xXdbba9frFj0=OqFfea0dXdd9vqai=hGuQ8kuc9pgc9s8qqaq=dirpe0xb9q8qiLsFr0=vr0=vr0dc8meaabaqaciaacaGaaeqabaqabeGadaaakeaacqWGjbqsdaWgaaWcbaGaemOyaiMaeyOeI0IaeGymaedabeaakiabg2da9iabdMgaPjabgEHiQmaalaaabaGaemiBaWMaeyOeI0ccbiGae8xBa0Mae8xAaKMae8NBa4MaeiikaGIaemiBaWMaeiilaWIaemyBa0MaeyOeI0IaeGimaaJaeiOla4IaeGynauJaey4fIOIaemyzauMaeiykaKcabaGaemyzaugaaiaaxMaacaWLjaWaaeWaaeaacqaIYaGmaiaawIcacaGLPaaaaaa@4B58@

Ib=i∗min(r,m+0.5∗e)−max(l,m−0.5∗e)e     (3)
 MathType@MTEF@5@5@+=feaafiart1ev1aaatCvAUfKttLearuWrP9MDH5MBPbIqV92AaeXatLxBI9gBaebbnrfifHhDYfgasaacH8akY=wiFfYdH8Gipec8Eeeu0xXdbba9frFj0=OqFfea0dXdd9vqai=hGuQ8kuc9pgc9s8qqaq=dirpe0xb9q8qiLsFr0=vr0=vr0dc8meaabaqaciaacaGaaeqabaqabeGadaaakeaacqWGjbqsdaWgaaWcbaGaemOyaigabeaakiabg2da9iabdMgaPjabgEHiQmaalaaabaacbiGae8xBa0Mae8xAaKMae8NBa4MaeiikaGIaemOCaiNaeiilaWIaemyBa0Maey4kaSIaeGimaaJaeiOla4IaeGynauJaey4fIOIaemyzauMaeiykaKIaeyOeI0Iae8xBa0Mae8xyaeMae8hEaGNaeiikaGIaemiBaWMaeiilaWIaemyBa0MaeyOeI0IaeGimaaJaeiOla4IaeGynauJaey4fIOIaemyzauMaeiykaKcabaGaemyzaugaaiaaxMaacaWLjaWaaeWaaeaacqaIZaWmaiaawIcacaGLPaaaaaa@5787@

Ib+1=i∗max(r,m+0.5∗e)−re     (4)
 MathType@MTEF@5@5@+=feaafiart1ev1aaatCvAUfKttLearuWrP9MDH5MBPbIqV92AaeXatLxBI9gBaebbnrfifHhDYfgasaacH8akY=wiFfYdH8Gipec8Eeeu0xXdbba9frFj0=OqFfea0dXdd9vqai=hGuQ8kuc9pgc9s8qqaq=dirpe0xb9q8qiLsFr0=vr0=vr0dc8meaabaqaciaacaGaaeqabaqabeGadaaakeaacqWGjbqsdaWgaaWcbaGaemOyaiMaey4kaSIaeGymaedabeaakiabg2da9iabdMgaPjabgEHiQmaalaaabaacbiGae8xBa0Mae8xyaeMae8hEaGNaeiikaGIaemOCaiNaeiilaWIaemyBa0Maey4kaSIaeGimaaJaeiOla4IaeGynauJaey4fIOIaemyzauMaeiykaKIaeyOeI0IaemOCaihabaGaemyzaugaaiaaxMaacaWLjaWaaeWaaeaacqaI0aanaiaawIcacaGLPaaaaaa@4B62@

When *e *= 0, this model regresses to the model of direct binning used by NoDupe [10], except that it only used the most significant peaks for binning. While it is characterized by the same computation complexity and memory storage requirement as the direct binning, the profiling alleviates the problem in the event that the peaks are located in the neighboring bins due to measurement errors.

### Similarity measures

The resulting spectrum profiles are represented as vectors of intensity in each bin. The problem of comparing spectra is reduced to determining the similarity of the corresponding profiles. The intensities of peaks in two spectra may still be on different scales even when their deviations are stabilized. Therefore, it is not appropriate to use a direct measure of the geometric distance, such as Euclidean distance (*L*_2_-norm), between a pair of spectra. In this study, we considered three options.

1. The correlation coefficient in Eqn 5 is a well-founded statistical method to evaluate the similarity between two vectors, as used in SEQUEST [5].

ρ(X,Y)=(X−μX)⋅(Y−μY)σXσY     (5)
 MathType@MTEF@5@5@+=feaafiart1ev1aaatCvAUfKttLearuWrP9MDH5MBPbIqV92AaeXatLxBI9gBaebbnrfifHhDYfgasaacH8akY=wiFfYdH8Gipec8Eeeu0xXdbba9frFj0=OqFfea0dXdd9vqai=hGuQ8kuc9pgc9s8qqaq=dirpe0xb9q8qiLsFr0=vr0=vr0dc8meaabaqaciaacaGaaeqabaqabeGadaaakeaaiiGacqWFbpGCcqGGOaakcqWGybawcqGGSaalcqWGzbqwcqGGPaqkcqGH9aqpdaWcaaqaaiabcIcaOiabdIfayjabgkHiTiab=X7aTnaaBaaaleaacqWGybawaeqaaOGaeiykaKIaeyyXICTaeiikaGIaemywaKLaeyOeI0Iae8hVd02aaSbaaSqaaiabdMfazbqabaGccqGGPaqkaeaacqWFdpWCdaWgaaWcbaGaemiwaGfabeaakiab=n8aZnaaBaaaleaacqWGzbqwaeqaaaaakiaaxMaacaWLjaWaaeWaaeaacqaI1aqnaiaawIcacaGLPaaaaaa@4EEE@

where *μ*_*X *_and *μ*_*Y *_are the means of *X *and *Y*, *σ*_*X *_and *σ*_*Y *_are their standard deviations, respectively. If the precursor ions of two spectra do not have exactly the same mass, the lengths of corresponding profiles *X *and *Y *are made the same by appending a few zeros to the shorter one. Correlation coefficients have the advantage of yielding values restricted to the range of [-1, 1]. Intuitively, spectral profiles of the same peptides are anticipated to return high and positive correlation coefficients.

2. The cosine value of spectral contrast angle given in Eqn 6 and its dot product variant, which are alternatives to the correlation coefficients, have been used to characterize the pairwise similarity in many other studies [10,17,19,20].

cos(X,Y)=X⋅Y‖X‖‖Y‖     (6)
 MathType@MTEF@5@5@+=feaafiart1ev1aaatCvAUfKttLearuWrP9MDH5MBPbIqV92AaeXatLxBI9gBaebbnrfifHhDYfgasaacH8akY=wiFfYdH8Gipec8Eeeu0xXdbba9frFj0=OqFfea0dXdd9vqai=hGuQ8kuc9pgc9s8qqaq=dirpe0xb9q8qiLsFr0=vr0=vr0dc8meaabaqaciaacaGaaeqabaqabeGadaaakeaaieGacqWFJbWycqWFVbWBcqWFZbWCcqGGOaakcqWGybawcqGGSaalcqWGzbqwcqGGPaqkcqGH9aqpdaWcaaqaaiabdIfayjabgwSixlabdMfazbqaamaafmaabaGaemiwaGfacaGLjWUaayPcSdWaauWaaeaacqWGzbqwaiaawMa7caGLkWoaaaGaaCzcaiaaxMaadaqadaqaaiabiAda2aGaayjkaiaawMcaaaaa@4833@

As with the correlation coefficient, the cosine similarity measure is theoretically restricted to [-1,1]. However, all the elements of the profile vector are non-negative, so the cosine values are effectively limited to the range [0, 1].

3. Another straightforward measure is to count the number of shared peaks in two spectra. Given two spectra with *m *and *n *peaks respectively, and assume that they share *k *peaks with same *m/z *values, the ratio of share peaks is calculated as

R=2∗km+n     (7)
 MathType@MTEF@5@5@+=feaafiart1ev1aaatCvAUfKttLearuWrP9MDH5MBPbIqV92AaeXatLxBI9gBaebbnrfifHhDYfgasaacH8akY=wiFfYdH8Gipec8Eeeu0xXdbba9frFj0=OqFfea0dXdd9vqai=hGuQ8kuc9pgc9s8qqaq=dirpe0xb9q8qiLsFr0=vr0=vr0dc8meaabaqaciaacaGaaeqabaqabeGadaaakeaacqWGsbGucqGH9aqpdaWcaaqaaiabikdaYiabgEHiQiabdUgaRbqaaiabd2gaTjabgUcaRiabd6gaUbaacaWLjaGaaCzcamaabmaabaGaeG4naCdacaGLOaGaayzkaaaaaa@39A2@

Since the intensity information is completely ignored, we hypothesize that this measure will be less discriminating than the above measures in distinguishing spectra.

### Models for reference spectra

There are various ways to generate a reference spectrum for each peptide. The most direct approach is to use an individual experimental spectrum. In our study, we chose the spectrum with the highest MASCOT score as the reference since it presumably reflects the real fragmentation pattern of a peptide better than low quality spectra. Alternatively, the theoretical spectra could also be used as references. Theoretical spectra are based on the simplified assumptions of the peptide fragmentation pattern. However, such a model does not accommodate instrument- and peptide-specific contributing factors during the fragmentation. Consequently, the experimental spectra may display limited resemblance to the corresponding theoretical spectra. Therefore, we expected that the experimental spectra for a peptide would be more similar to sibling spectra from the same peptide than to its theoretical counterpart.

Nevertheless, different MS/MS spectra from the same peptide may not always be similar due to a variety of factors. First, different mass spectrometers have their own sensitivity and measurement accuracy. Second, spectral generation is subject to many variables such as signal to noise ratio and variations in collision energy. Furthermore, preprocessing software, such as MASCOT Distiller, may alter the quality of spectra derived from the raw data. Therefore, using a single spectrum as a reference may be fragile. Indeed, it has already been observed that some spectra from the same peptides had very low pairwise similarity. It is thus desirable to find a more robust and stable reference to represent the peptide when a group of spectra for the same peptide are available. Pep-Miner [17] introduced a method to generate a representative for a spectrum cluster which involved a sophisticated procedure to merge peaks, filter noise and identify the significant peaks. Another software MS2grouper [18] adopted a greedy algorithm to iteratively select the most significant peaks to construct a representative spectrum. In this study, we considered the more straightforward approach of computing the ensemble average. If a group of spectra are assigned to the same peptide, all the peaks in each spectrum are merged into a new spectrum. Then the intensity of each peak in this new spectrum is divided by the group size. Intuitively, the peaks from the most abundant ions will be emphasized and retain relatively high intensities. We expected that such reference spectra represent the essential patterns of ions for given peptides. Such spectra are referred to as the average spectra. Another possibility would be to take the highest similarity to a spectrum of a cluster. Formally, given a set of spectra {*s*_1_, *s*_2_,..., *s*_*k*_} from the same peptide and a new spectrum *s*', the similarity between each pair of spectra (*s*_*i*_, *s*'), *i *= 1,..., *k*, is calculated, and the highest one is assigned as the similarity score. The idea is similar to the classic nearest neighbor approach for classification problems. To avoid confusion, such spectra were referred to as the closest neighbors. In our experiments we compared the performances of the four alternatives described above.

## Competing interests

The author(s) declare that they have no competing interests.

## Authors' contributions

JL developed the methods, conducted the experiments. AWB and JM provided the biological samples for peptide identification. CMY, BC and CEHB participated in the software development, discussion, and proofreading of the manuscript. REK supervised and coordinated the project. JL and REK wrote the manuscript. All authors read and approved the final manuscript.
